# An Efficient Clinical Decision Support Framework Using IoMT Based on Explainable and Trustworthy Artificial Intelligence with Transformer Model and Blockchain-Integrated Chunking

**DOI:** 10.3390/diagnostics16010007

**Published:** 2025-12-19

**Authors:** Kübra Arslanoğlu, Mehmet Karaköse

**Affiliations:** 1 The Department of Software Engineering, The University of Firat, 23119 Elazig, Turkey; 2 The Department of Computer Engineering, The University of Firat, 23119 Elazig, Turkey; mkarakose@firat.edu.tr

**Keywords:** internet of medical things, cloud, edge, artificial intelligence, blockchain, explainability, trustworthy

## Abstract

**Background/Objectives:** The use of edge–cloud architectures has increased rapidly to move the analysis of AI-enabled health data to global environments. However, data security, communication overhead, cost-effectiveness, and data transmission losses are still important problems to be solved. **Methods:** In this paper, we propose a reliable, explainable, and energy-efficient stress detection framework supported by a cost-oriented blockchain-based content-defined chunking approach to minimise the losses during data transfer. In the proposed architecture, the Nurse Stress dataset represents IoMT data. While the chunking process reduces communication volume and storage costs by avoiding data duplication, blockchain technology eliminates the risks of unauthorised access and manipulation by ensuring the immutability and traceability of data blocks. **Results:** All Transformer-based models have demonstrated over 99% accuracy. The TimesNet model, in particular, has been designated as the system’s reference model, exhibiting superior performance in terms of both stability and accuracy. The main contribution of this study lies in proposing one of the first integrated frameworks that jointly employs chunking-based data management, blockchain-enabled trust mechanisms, and edge–cloud computing with XAI to ensure secure and transparent IoMT data processing. The proposed system not only performs highly accurate stress detection, but also optimises the dimensions of reliable data transmission, energy and cost efficiency, and clinical reliability. **Conclusions:** In this respect, the study presents a scalable, reliable, and repeatable approach in health decision support systems by combining data security, integrity, and explainability issues, which are addressed separately in the literature, in a holistic manner.

## 1. Introduction

The concept of the Internet of Things (IoT) greatly expands people’s ability to access information, while cloud computing brings powerful information processing capability to IoT technology and improves the efficiency of IoT systems [1]. Internet of Medical Things (IoMT) is an IoT-based technology that is generally used for the development of IoT-enabled healthcare systems for monitoring various vital signs such as ECG, pulse, blood pressure, and brain signals [2]. Nowadays, IoMT is a technology that has the potential to revolutionise the development of the healthcare industry. IoMT offers a system that significantly improves the quality, accessibility, and personalization of healthcare services by connecting medical devices and their applications over the internet [3,4]. These smart devices, which continuously monitor the health status of patients and collect data of their health status, form the basis for real-time health monitoring and interventions. However, for this technology to realise its full potential, the collected data needs to be processed and analysed effectively. This is a significant challenge, especially given the large data volumes and complex data structures [5].

Cloud and edge computing technologies provide solutions for processing the large amounts of data collected in IoMT systems. Cloud computing enables centralised processing of health data with powerful data processing capacity and large storage space, while edge computing enables data processing on the devices themselves, reducing latency and easing the burden on data transmission [6]. The integration of these two technologies has the potential to enhance the effectiveness of IoMT in smart healthcare systems and enable faster, more accurate clinical decision-making. By leveraging artificial intelligence (AI), real-time health monitoring, disease prediction, and prevention can be achieved with improved user experience and system reliability [7]. Blockchain is an ideal technology to significantly improve the functionality and security/privacy of the cloud ecosystem thanks to its superior properties such as decentralisation, immutability, traceability, anonymity, and transparency. Blockchain and cloud computing integration has the potential to improve efficiency in authentication and access control, data security and privacy, transaction fairness, decentralised data sharing, and supply chain management applications [8]. Blockchain is an innovative technology that provides a distributed ledger of data to nodes according to authentication and security rules. The main purpose of blockchain used in IoMT systems is to ensure data integrity and security by minimising the risk of tampering or unauthorised access of data on any node [9].

Traditional offloading schemes may be insufficient to meet the demands of changing network environment and low latency. AI techniques, especially deep learning and reinforcement learning, have provided significant breakthroughs in this area and researchers have developed resource allocation schemes based on deep reinforcement learning for cloud–edge systems [10]. A service deployment methodology that takes security into account should enable decision makers to understand why a particular deployment is best and, therefore, XAI techniques have become of increasing interest to the security community for different stakeholders such as patients, doctors, and regulatory authorities because the recommendations they provide are understandable and provable [11,12,13,14,15,16].

With the proliferation of cloud-based services, data security and management has become one of the most important issues. To solve this problem, encryption techniques are widely used to protect sensitive data. Secure cloud-based data shredding and file encryption system mainly focus on the development of a file encryption and shredding system within cloud computing infrastructure [17]. The increasing reliance on third-party cloud providers to store data, especially in the healthcare field, has made it critical to guarantee the security, integrity, and accessibility of information. Since traditional data protection techniques are no longer sufficient, chunking-based approaches have been proposed as effective solutions against cyber threats [18]. Moreover, advanced chunk-based data fragmentation frameworks can reduce both transmission and storage overhead by combining fingerprinting, fragmentation, and encryption techniques [19]. In addition, developments in the field of AI also support this structure. The emergence of the Transformer model has demonstrated its powerful capabilities for processing temporal data, and applications have developed in the field of multivariate time series. The Transformer model processes sequence data in parallel and can extract internal information within the sequence, using the self-attention mechanism to compute dependencies and correlations between different sequence points [20].

In this study, we propose a framework that enables secure, explainable, cost-effective, and energy-efficient processing of IoMT data. The proposed approach combines data pre-processing on edge devices, model training in cloud environment, security with blockchain, data load reduction with chunking, and time series analysis with Transformer. Experimental results show that significant gains are achieved in terms of loading time, verification time, communication volume, and cost. Furthermore, the high accuracy of the Transformer-based model and the explainability with SHAP show that the proposed framework offers a strong potential as well as technical and clinical accuracy. Healthcare systems are facing serious challenges due to increasingly heterogeneous medical data and the urgent need for secure, cost-effective, and real-time decision support. Although recently developed IoMT frameworks improve healthcare, existing studies mostly focus on non-integrated single-structure technologies and mainly emphasise accuracy performance. Critical aspects such as communication efficiency, energy consumption, interpretability, and end-to-end security are often ignored in these studies. To address these shortcomings, we propose a novel edge–cloud framework based on blockchain and chunking. The importance of the study is highlighted by the following contributions:

- This research enables secure, reliable, and efficient transmission of health data by integrating content-defined chunking and blockchain technologies together for the first time in an edge–cloud AI architecture.
- The fragmentation approach, combined with blockchain integrity verification, prevents the acceptance of faulty or incomplete data blocks, thereby reducing the retry rate and associated transmission costs.
- While in the literature chunking is only used for deduplication purposes, in this study the chunking approach is implemented as a transmission module that optimises the reliable transmission of IoMT stress data. Combined with blockchain logging, both verifiable reliability and traceable integrity are ensured.
- This paper aims to achieve higher accuracy and training efficiency compared to classical machine learning and deep learning approaches by using a Transformer-based model for multivariate time series stress detection within the proposed architecture.
- SHAP-based explainability methods have increased the confidence of health providers by making clinical estimates transparent and understandable.
- The proposed architecture offers significant contributions in real-time stress detection and clinical decision support, which are critical for scalable, secure, and interpretable IoMT-based decision support systems.

In this architecture, health data is pre-processed on edge devices, split into optimised chunks, transmitted to the cloud, validated by blockchain, and processed by Transformer-based models. This design not only improves accuracy but also reduces latency, energy consumption, and operational costs. In addition, it guarantees security and transparency. The main contribution of this work is to combine chunking, blockchain, edge–cloud computing, and explainable artificial intelligence (XAI) in a single framework, which offers both technical efficiency and clinical reliability with real-time proven values. Thus, it is a clear departure from previous studies that addressed the dimensions in these areas separately.

## 2. Proposed Method

**Figure 1:**
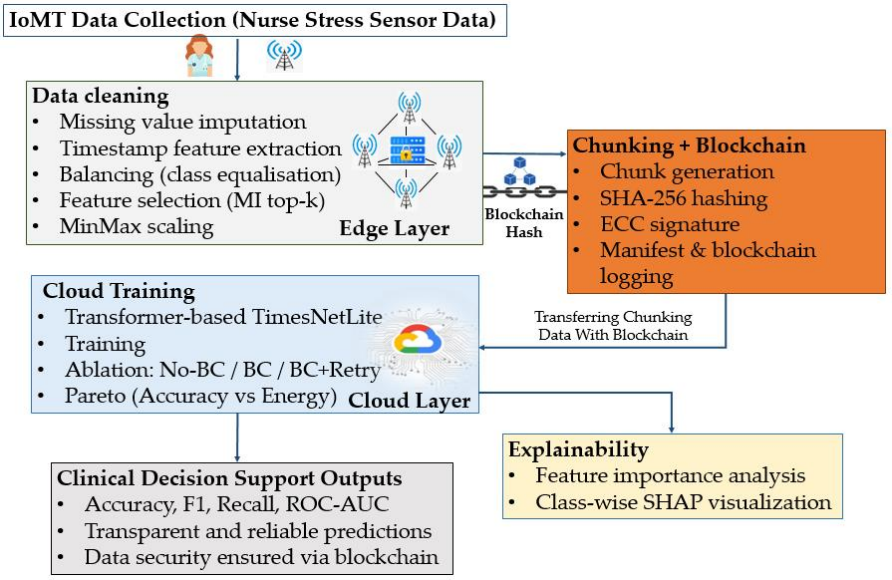
General structure of the proposed approach.

In this section, the main components and architecture of the proposed method are presented in detail. In our work, we propose an edge–cloud-based, blockchain- and chunking supported, Transformer-based stress detection framework that enables secure, efficient, and explainable processing of IoT-based health data. The proposed methodology is given in Figure 1.

According to the general structure of the study, for which the flow chart is given in Figure 1, it consists of the following stages:

1. Data acquisition from IoT devices: The Nurse Stress dataset was used to simulate the IoMT ecosystem in healthcare [21]. This dataset contains multivariate sensor data reflecting stress levels. The data was first imported into the edge device.
2. Pre-processing and feature selection on the edge device: The data were processed on the edge device, unnecessary columns were removed, and dimensionality reduction (PCA) and data balancing (SMOTE) methods were applied. Thus, the communication load was reduced and data imbalances that reduce the model performance were eliminated.
3. Blockchain and chunking integration: The data was divided into small pieces by the content-defined chunking method. The hash value of each chunk was generated and stored on the blockchain, thus preventing data duplication and ensuring data integrity, immutability, and traceability. Chunk sizes and hash calculations were recorded experimentally, and these parameters were then used to calculate the communication and storage overhead.
4. Secure transfer to the cloud: The data processed on the edge device and validated with the blockchain was securely transferred to the cloud environment. The integrity of the transmitted data is guaranteed by checking the compatibility of the hash values.
5. Transformer-based model training: Transformer-based deep learning models with high capacity to analyse multivariate time series were trained in the cloud environment.
6. Integration of XAI: SHAP was integrated to ensure transparency of the model outputs. Thus, clinicians and end-users can interpret the features on which the model’s decisions are based.
7. Cost- and energy-efficiency calculations: To evaluate the practical applicability of the system, measurements such as upload time, verification time, communication volume, chunk sizes, blockchain verification times, and energy consumption (Joule) were performed.

All experimental implementations of the system developed in this study were carried out in a Google Colab Pro+ environment with Python 3.10 language. NVIDIA T4 GPU (16 GB VRAM), 12 GB RAM, and Intel Xeon 2.20 GHz CPU resources were used in the experiments. The modelling process was performed with PyTorch (v2.2), Scikit-learn (v1.4), SHAP (v0.45), NumPy (v2.0.2), Pandas (v2.2.2), Matplotlib (v3.10.0), Seaborn (v0.13.2). Edge–cloud architecture was implemented in a simulation-based environment instead of real hardware; data processing stages were performed at the edge level and model training was performed at the cloud level. For blockchain integration, SHA-256-based hash function, digital signature verification process with ECC (Elliptic Curve Cryptography), and the Python hashlib module were used.

### 2.1. Dataset and IoMT Context

IoMT has facilitated patient follow-up and accelerated diagnosis and treatment processes in healthcare through sensors, wearable devices, and remote monitoring technologies [22,23]. These systems, which increase access to healthcare services, especially in less developed regions, are supported by low-energy technologies [24]. Today, low-latency, real-time data transfer is possible with 5G/6G connection infrastructures, which makes it possible to integrate IoMT systems with cloud–edge structures [25]. However, the success of integrating this structure into systems depends on effectively addressing factors such as data security, privacy, big data management, and system scalability [22]. IoMT devices are classified as wearable devices, home healthcare technologies, and in-hospital smart equipment [26,27,28].

In the literature, many studies have been carried out to analyse IoMT data. For example, Wu et al. [17] proposed AI-based architectures for cloud–edge integration in IoT applications. Wang et al. [18] developed a cloud–edge-based platform for managing the complexity of cyber–physical–social systems. In another study examining the role of 5G and IoMT in health diagnostics processes, it was emphasised that big data analytics are empowered by federated learning [20]. In addition, Ding et al. [29] proposed deep-learning-based cognitive services in both the cloud and edge layer with a CloudCNN–EdgeCNN structure. In a study on in-home health monitoring, privacy-preserving personalised models were developed using federated learning [30]. In the PRE-ACT project, federated learning and mobile applications were integrated to predict radiotherapy-related side effects [31].

In this study, a dataset based on multimodal physiological signals, originally published by Hosseini et al. in the Dryad repository and added to the Kaggle platform, was used to indicate nurses’ stress levels. The dataset was obtained from 15 female nurses aged between 30 and 55 during regular hospital shifts. Consisting of approximately 11.5 million timestamped records, the dataset has 9 attributes: X, Y, Z, EDA, HR, TEMP, id, datetime, and label. Empatica E4 wearable sensors were used to continuously measure electrodermal activity (EDA), heart rate (HR), and skin temperature (TEMP) during data collection; simultaneously, data from a three-axis accelerometer (X, Y, Z) tracking body movement and orientation were recorded. Each record is labelled with one of three stress levels—low, normal, or high and associated with a unique anonymous participant ID. The study was approved by the University Institutional Ethics Committee (FA19–50 INFOR), and informed consent was obtained from all participants prior to data collection. The dataset provides a comprehensive foundation for research in the areas of continuous stress detection, workload analysis, and explainable artificial-intelligence-based modelling. This multidimensional and time-series-based dataset represents real-world IoMT scenarios. Its characteristics lend themselves to use on edge devices for pre-processing, feature selection, and balancing, reflecting the need to process high-dimensional, heterogeneous health data before it is transferred to the cloud.

Table 1 provides details of studies conducted using the same dataset. In the study by Abu-Samah et al., the data was processed in a TinyML-based framework to work in embedded systems, data imbalance was corrected using the NearMiss-1 method, and the XGBoost model was embedded on Raspberry Pi RP2040, achieving approximately 86% accuracy [32]. In the study by Chauhan and Singh, the same dataset was evaluated within the scope of stress analytics, EDA and HR signals were identified as the strongest predictors, and accuracy rates of 83–90% were reported using KNN and Random Forest algorithms [33]. Liu, Xue, and Hou, focusing on data privacy, proposed a federated learning (FL)-based approach, combined local neural network models using the FedAvg algorithm, and achieved over 90% accuracy [34]. These studies demonstrate that models developed using the same dataset offer high accuracy and generalisability under different computational paradigms. Although these studies use the same Nurse Stress dataset, their pre-processing steps, train–test split ratios, normalisation strategies, and validation protocols differ significantly.

### 2.2. Cloud–Edge and Blockchain-Chunking Framework for Stress Data

In this study, the Nurse Stress dataset obtained from Kaggle was used to analyse the stress levels of nurses based on their physiological signals. This multidimensional and time-series-based dataset represents real-world IoMT scenarios. Its characteristics lend themselves to use on edge devices for pre-processing, feature selection, and balancing, reflecting the need to process high-dimensional, heterogeneous health data before it is transferred to the cloud. The deduplication process consists of four basic stages, chunking, fingerprinting, indexing and writing. There are two types of chunking operations; file-level chunking offers low deduplication rate as it treats the whole file as a single piece, while block-level chunking can be implemented in fixed-size (FSC) or content-defined (CDC) format and provides higher efficiency by users [35,36]. Recent studies show that chunking-based data processing approaches play a critical role not only for storage purposes, but also for secure data transfer, computational cost reduction, and blockchain performance optimisation. Among the studies, SmartChunk [37] developed a hash-based hybrid content-defined chunking (CDC) method to achieve a high compression ratio and lower duplicate data rate. In another study using a similar encryption process, privacy and access control were achieved by encrypting each chunk independently in a cloud environment [38]. Similarly, a study proved that chunk size has a direct impact on blockchain chain size, verification time, and transaction cost [39]. In the field of healthcare, Subramani and Jothi proposed a chunk-based RAID encryption model to optimise data privacy and scalability in blockchain networks [40]. In addition, the study with blockchain optimisation reduced ledger size and increased verification performance with adaptive compression and advanced data structures [41]. In another study with chunk–cloud, SLO, and input data, size-aware dynamic chunk configurations reduced costs by up to 61% [42].

On the other hand, the need for real-time data processing in healthcare applications has increased the importance of cloud–edge architectures. The processes applied at the edge layers process data close to the source, shortening response times and reducing the pressure on network bandwidth [43]. Larger, complex, and time-consuming tasks are executed on the cloud side to utilise scalable resources [44,45,46,47]. Cloud architecture offers a flexible structure for centralised storage, analysis, and data sharing with different systems. Cloud architecture provides a variety of operations with three basic service models, SaaS, PaaS, and IaaS [48,49,50,51]. Thanks to this structure, end-to-end total processing time, edge, cloud, and communication delays can be calculated over the components and speed and efficiency can be provided together in decision-making processes [52,53].

Recent research on the deep learning side also shows that chunking is critical not only for storage but also for memory and computational efficiency. Some of these studies have reduced activation memory by over 80% in long-sequence inference and increased the maximum sequence length up to three times with only 10% speed loss [54]. The masked chunk processing technique reduced GPU memory usage by more than three times, was able to process long audio inputs up to 16 h, and improved the word error rate by up to 7.7% [55]. These results clearly show that the chunk-based strategies we favoured in our study are superior to alternative methods in both storage and computational efficiency. The proposed Cloud–Edge and Blockchain-Chunking Framework for Stress Data model, unlike the studies in the literature, combines chunking and blockchain integration with a focus on XAI and performance–security balance for health data. Thus, it makes a unique contribution to IoMT-based clinical decision support systems in terms of both accuracy and reliable data transmission.

**Figure 2:**
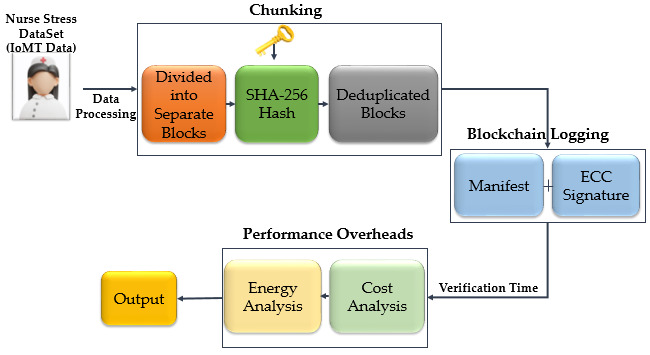
Proposed framework showing data chunking, blockchain logging, and performance overhead analysis for stress data.

In the dimension of integration with blockchain, in systems that provide secure sharing of IoT data with multi-level access control, blockchain increases security not only with data encryption but also with immutability, traceability, and transparency [56,57,58,59,60,61]. In addition, the basic cryptographic building blocks used in the blockchain system include public-key cryptography, zero-knowledge proof techniques, and SHA-256-based hash functions. These structures guarantee the integrity of the chain by creating a Merkle tree [62,63]. Figure 2 shows the architecture of the proposed system, which integrates chunk-based data splitting, blockchain logging, and performance–cost analysis modules to ensure secure, efficient, and traceable processing of stress data.

In the proposed work, chunk–blockchain structure is integrated with cloud–edge architecture for secure and efficient processing of stress data. While the edge layer performs low latency pre-processing tasks, the cloud layer performs more complex model training and XAI applications. Blockchain integration ensures that the data is protected with immutability, integrity, and traceability features. Although the main reason for preferring chunk-based methods is to increase storage/memory efficiency, when used in conjunction with blockchain, it offers a reliable, scalable, and cost-efficient framework. The mathematical formulation of the chunk + blockchain mechanism, which is the most important part of the study, is given in Equations (1)–(5) [40,64,65].

(1) $$D=\overset{n}{\underset{i=1}{⋃}}\;C_{i}$$

D: The entire dataset.

*n*: Total number of chunks.

*C_i_*: *i*th chunk of data.

The data stream is fragmented into small pieces. This process enables the detection of repetitive data blocks.

(2) $$H_{i}=SHA-256\left(C_{i}\right)$$

(3) $$E_{i}=AES_{k}\left(C_{i}\right)$$

*H_i_*: Hash value of the *i*th chunk

*E_i_*: Encrypted chunk data

k: Chunk encryption key

The data is divided into small chunks by fixed-size (FSC) or content-defined (CDC) algorithms. By hashing each part, repetitive parts are removed and storage and transmission costs are reduced.

(4) $$H_{\mathrm{manifest}\;}=SHA256\left(H_{1}\left\|H_{2}\right\|\ldots \|H_{n}\right)$$

(5) $$T=\left(H_{\mathrm{manifest}},\;\mathrm{meta}\right),\;B\leftarrow B\cup \{T\}$$

$H_{\mathrm{manifest}}$: Combined hash consisting of all chunk hashes

T: Transaction written to blockchain

meta: Additional information such as timestamp, user ID

*B*: Blockchain ledger

All hash values are combined to create a manifest hash. It is recorded on the blockchain and signed with ECC (Elliptic Curve Cryptography). All chunks are secured under a single hash by writing the manifest generated from all hash values to the chain.

Equation (6) defines the basic components that make up the total delay in the edge–cloud architecture. Thus, bottlenecks in the system can be analysed [63].

(6) $$L_{\mathrm{total}\;}=L_{\mathrm{split}\;}+L_{\mathrm{proc}\;}+L_{\mathrm{link}\;}+L_{\mathrm{verif}\;}$$

$L_{\mathrm{total}}$: Total system delay

$L_{\mathrm{split}}$: Chunking delay

$L_{\mathrm{proc}}$: Processing delay

$L_{\mathrm{link}}$: Communication (transmission) delay

$L_{\mathrm{verif}}$: Blockchain verification latency

In Equation (7), reloading rates and total cost calculation of the system in the case of chunk loss are given.

(7) $$U_{\mathrm{ploadCost}\;}=\;\mathrm{DatasetSize}\;(GB)\times \;\mathrm{CloudStoragePrice}$$

(8) $$\mathrm{ExtraCost}\;=\;\mathrm{RetryRatio}\;\times U_{\mathrm{ploadCost}\;}$$

$U_{\mathrm{ploadCost}}$: Cost of uploading data to the cloud

CloudStoragePrice: Cloud storage price per 1 GB

*ExtraCost*: Additional cost in case of chunk loss or reload during transmission

*RetryRatio*: Reload rate

Equations (9) and (10) show the calculation of total energy consumption for both model training and blockchain validation processes [66].

(9) Energy(J)= Power (W)× Time (s)

(10) $$E_{\mathrm{total}\;}=E_{\mathrm{train}\;}+E_{\mathrm{verify}\;}$$

Power(W): Average power consumption of the GPU (watts)

Time(s): Training time (seconds)

$E_{\mathrm{train}}$: Energy consumption during the training phase

$E_{\mathrm{verify}}$: Additional energy consumed during blockchain verification

These equations form the basis of the proposed system in terms of both data security and computational efficiency. The sharding and hash-based verification equations reduce the storage load that may occur by preventing data duplication, while metrics related to energy, cost, and verification times make it possible to evaluate the performance, reliability, and sustainability dimensions of the system. This structure expresses the theoretical infrastructure of the study as a mathematical representation of the integrated relationship between blockchain-supported secure data transfer and edge–cloud AI architecture.

### 2.3. Transformer Model for Sensor Data

Transformer architecture has surpassed classical sequential structures by providing the ability to model long-term dependencies with the self-attention mechanism first proposed by Vaswani et al. [67]. This approach has greatly improved both natural language processing and time series prediction with the advantage of parallel computation and the ability to capture the global context more effectively [68]. Especially in IoT- and IoMT-based applications, Transformer-based approaches have gained innovation as an important solution area in terms of processing high volumes of sensor data under significant constraints such as delay, energy, and data loss [69].

TimesNet is a time series Transformer derivative developed by Wu et al. Unlike Transformer models, TimesNet represents the data in “time-frequency” space instead of “time-space”. This structure processes time series in 2D variation format with convolution layers based on multiple periods. This basic component, called “TimesBlock”, can capture both short-term sudden changes and long-term physiological trends simultaneously. In the model setup, the TimesNet model was tested on financial series, energy consumption prediction, and health sensor data, and it was found to achieve 4–8% accuracy improvement compared to classical Transformer, LSTM, and Informer models [70]. In recent studies, Transformer architectures have been designed not only to improve prediction performance but also to meet critical requirements such as security, energy efficiency, and explainability.

For example, the Transformer model using hybrid encryption combines hybrid AES + ECC and Swin Transformer architecture for secure data transmission and anomaly detection in IoMT-based systems. The Greylag Goose Optimiser algorithm performs hyperparameter optimisation, achieving 97.3% accuracy and low energy consumption. In addition, this model reduces unnecessary repetition in health data by addressing the deduplication problem; in this respect, it is among the reference models with the blockchain-chunking-based data management strategy proposed in our study [71]. Similarly, FogMedX-Transform developed a Transformer-based fog-enabled IoMT framework that enables task sharing between cloud computing and edge devices, using a special attention mechanism that enables task interoperability. The study achieved high energy efficiency with 97.5% anomaly detection accuracy and 98.7% task interoperability [72].

Kalakoti and colleagues proposed an explainable Transformer-based architecture for attack detection in IoMT networks. SHAP- and LIME-based explainability modules were integrated into the system to make the model’s decisions interpretable. The Transformer model achieved an F1 score of 96.2% on the CICIoMT2024 dataset used in the study. Furthermore, it was reported that the model achieved a 7% increase in overall accuracy compared to classical CNN and LSTM approaches [73]. This study provides a fundamental reference for our proposed system, particularly in terms of presenting the theoretical foundation of the SHAP-based explainability layer. In this study, TimesNet, Autoformer, PatchTST, Non-Stationary Transformer (NST), and classical Transformer architectures were systematically evaluated under the same experimental conditions. All models were tested using the same data processing steps and evaluation metrics. Furthermore, a comprehensive comparison was conducted, taking into account computational efficiency indicators such as GPU memory usage and training time. TimesNet’s multi-scale convolutional filters provided higher accuracy than other Transformer-based approaches, as they effectively modelled both short-term stress fluctuations and long-term trends.

Table 2 shows the hyperparameters used in the proposed TimesNet-based stress detection model. All hyperparameters were kept identical to other Transformer-based models, thus ensuring that comparisons are repeatable and fair.

A single unified experimental protocol was applied to ensure that all models used in the study could be evaluated in a reproducible manner. The dataset was first processed by removing duplicate data and filling in missing values with average values. In the study, the dataset was divided into training, validation, and test subsets using a stratified sampling approach, with 70% for training, 20% for validation, and 10% for testing. A total of 25-fold cross-validation, consisting of 5 repetitions and 5 folds, was performed on the training subset. Mutual information-based feature selection was applied in each fold to determine the nine features with the highest information gain. The scaling process was fitted only in the training section of the relevant fold and applied in the validation section using these parameters to completely prevent data leakage. All models were trained with the same set of hyperparameters to ensure they were evaluated under equal conditions. After calculating the average performance values obtained from cross-validation, the final model was retrained on the entire training data, and the validation and test subsets, which had been set aside for performance evaluation, were used.

**Figure 3:**
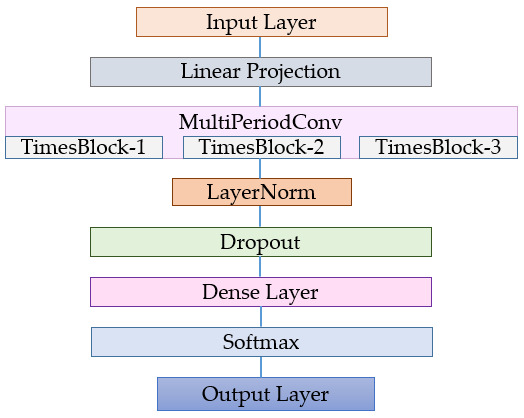
Architecture of the proposed TimesNet-based model.

The general structure of the TimesNet-based classification architecture, which is the most suitable model for time series among Transformer models and yields the highest performance results, is shown in Figure 3. The model offers a Transformer approach enhanced with multi-scale convolutional blocks to capture both short-term and long-term stress patterns. The model’s input layer directly receives the multi-variable sensor signals obtained from the Nurse Stress dataset. These signals are first passed through the Linear Projection layer and converted into higher-dimensional representations. Then, the MultiPeriodConv block, which forms the core of the feature extraction process, is activated. Within this block, successive TimesBlock layers perform multi-scale filtering on the data with different period lengths of 3, 5, 7, 9, and 11, successfully learning both sudden changes and long-term trends. The obtained intermediate representations are passed through a LayerNorm layer to increase statistical stability, followed by Dropout to reduce overfitting. The learned features are then transferred to the classification space within the Dense Layer, and the Softmax function calculates the probability of each stress level. In the final stage, the Output Layer produces the final stress class prediction.

The explainability analysis was conducted using the SHAP KernelExplainer method to clarify the data and model interpretability within the scope of the study. Reliability and cost analyses were performed through a blockchain-based chunking mechanism. Data blocks ranging from 64 to 1024 lines were controlled using SHA-256 hashing, manifest verification, ECC signature placeholders, and a retry probability of 0.05. Energy measurements were obtained in real time from GPU power values via NVIDIA-SMI whenever possible. This experimental protocol provided a fair, reproducible, and computationally efficient comparison environment. The aim of the study was to establish the TimesNet model as a reference model, as it demonstrated high accuracy on IoT data, particularly due to its temporal awareness, showing higher accuracy and stability compared to other architectures.

## 3. Experimental Results

In this section, we present the performance results, explainability analyses, and blockchain-chunking-based reliability performance values of different Transformer-based models used in our study.

### 3.1. Model Comparison and Selection

All Transformer-based architectures used in the study were evaluated under the same training protocol with a balanced dataset, MI-based feature selection, and common hyperparameters. To measure the generalisation performance of the models more reliably, a 5 × 5 repeated stratified cross-validation approach was applied, and the mean and standard deviation values of the accuracy, precision, sensitivity, F1 score, and ROC-AUC metrics were calculated for each model. The results obtained are presented in Table 3.

Upon examining Table 3, it is observed that all models achieved accuracy values above 99%, and Transformer-based approaches demonstrated high generalisation success in the stress classification task. Among the models, TimesNet is the most successful architecture of the study, demonstrating the highest performance across all metrics. In particular, it offers an extremely balanced classification performance with 99.6% accuracy, precision, sensitivity, and F1 score. The ROC-AUC value of 99.8 indicates that the model has a very high discrimination power between classes. Although the PatchTST and TransformerEncoder models performed well after TimesNet, TimesNet’s multi-scale convolution structure allows it to model short- and long-term patterns more effectively. Although the Autoformer and NST models also achieved high accuracy levels, their performance is relatively lower compared to other models. The performance of the models on a 20% validation and 10% test set was evaluated, and the results are presented in Table 4 based on metrics.

**Figure 4:**
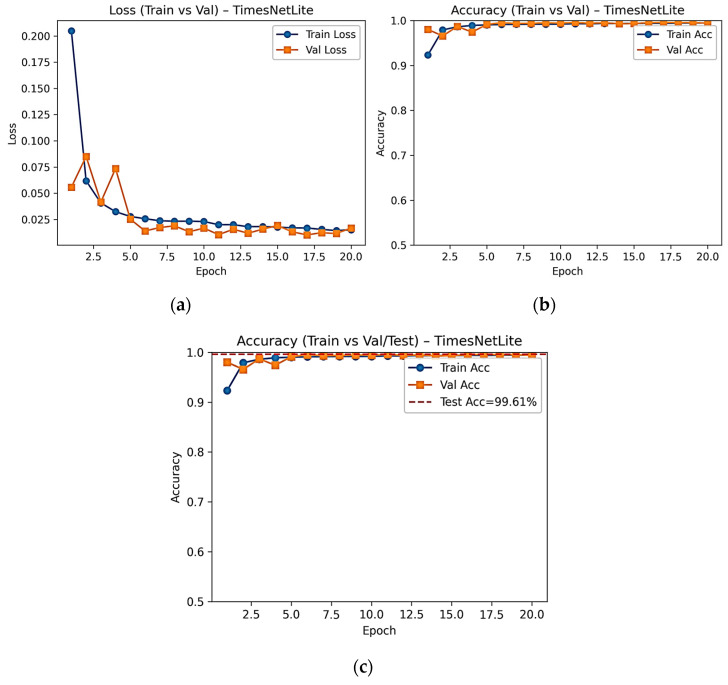
Training and validation loss (**a**), performance (**b**), and training–validation–test accuracy (**c**) curves of the TimesNet model.

Upon examining Table 4, it is observed that all Transformer-based models demonstrate extremely close and high performance on both the 20% validation and 10% test sets. The fact that all models achieve accuracy, precision, sensitivity, and F1 scores above 99% indicates that the data processing pipeline and training protocol used are functioning reliably. Within this close performance distribution, TimesNet ranks slightly ahead of other models in all metrics and maintains a stable superiority in classification success thanks to its multi-scale convolutional structure, which more effectively models short- and long-term patterns. Overall, the validation and test results support each other, indicating that the models have high generalisation capabilities and supporting the selection of TimesNet as the reference model. Figure 4 shows the loss and accuracy curves for the training process of the TimesNet model, as well as the validation and test set performances. The graphs show that the model exhibits a steady decrease in both training and validation losses and a rapid increase in accuracy values over 20 epochs.

**Figure 5:**
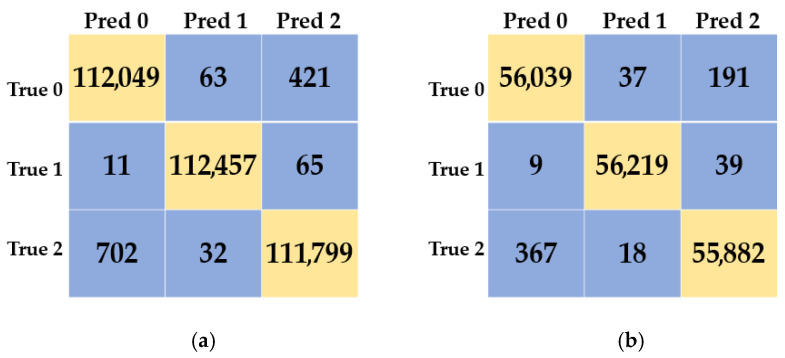
TimesNet model’s validation set confusion matrix (**a**), test set confusion matrix (**b**).

Figure 5 details the classification success of the TimesNet model on both the validation and test datasets. The confusion matrix on the validation set demonstrates the model’s generalisation ability during the learning process, while the test set matrix shows how the actual performance holds up on independent data. High accuracy rates in both matrices confirm that the model delivers consistent and reliable performance.

### 3.2. Explainability Analysis

**Figure 6:**
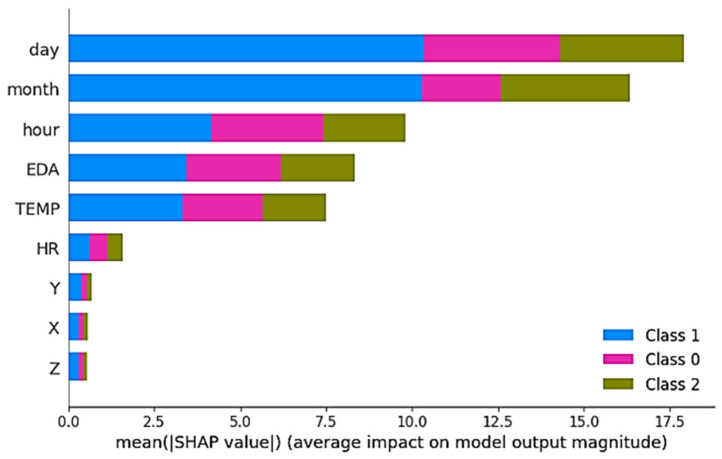
TimesNet model SHAP attribute importance ranking.

In order to improve the interpretability of the proposed model, SHAP (SHapley Additive exPlanations) analysis was applied to determine the contribution of each attribute to the model predictions, especially for clinicians. Figure 6 shows the attribute importance ranking based on the SHAP values obtained for the TimesNet model.

According to Figure 6, day, month, and hour variables have the highest impact on the time dimension, while physiological parameters, especially EDA (skin conductance) and temperature (TEMP), play a decisive role in model decisions. Heart rate (HR) and accelerometer axes (X, Y, Z) made lower contributions. The colours show the influence of the attributes for different classes (Class 0-low stress, Class 1-moderate stress, Class 2-high stress). These findings suggest that the model’s decision-making mechanism can be explained on biological grounds by taking into account both environmental characteristics and physiological signals in stress predictions.

**Figure 7:**
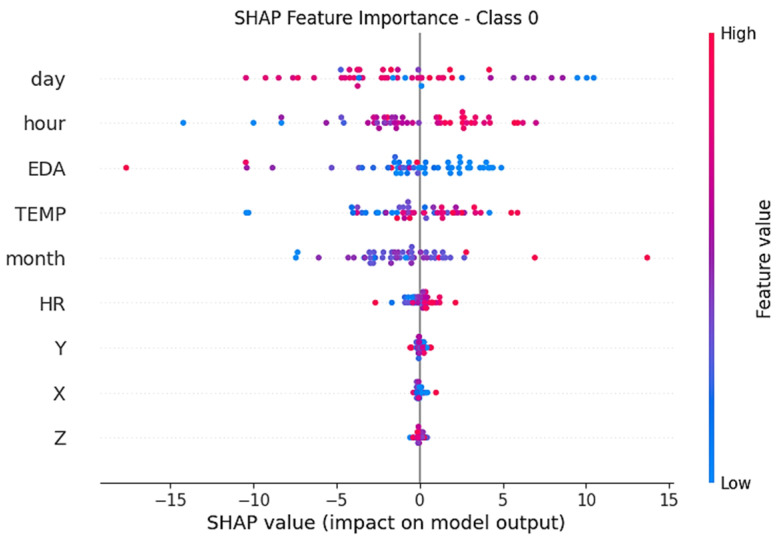
SHAP values for the TimesNet model’s Class 0 (low stress).

Figure 7 shows the attribute importance distributions of the TimesNet model over Class 0 (low stress) SHAP values. While time dimension attributes such as day, month, and hour are prominent, it is seen that especially EDA and TEMP values of physiological signals make a significant contribution to low stress classification. Low values of EDA are strongly reflected in the decision mechanism in this class.

**Figure 8:**
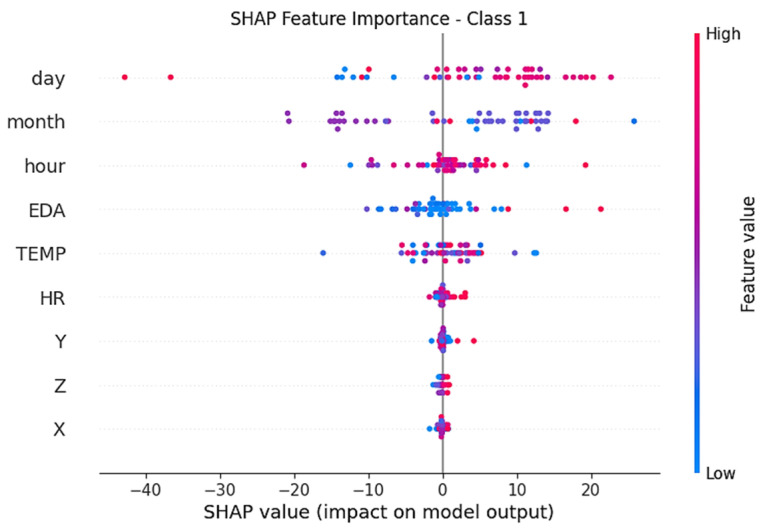
SHAP values for the TimesNet model’s Class 1 (moderate stress).

Figure 8 shows the attribute importance distributions of the TimesNet model over Class 1 (moderate stress) SHAP values. In this class, both time attributes (month, day, hour) and physiological attributes (EDA, TEMP) play a decisive role together. Especially high values of EDA and TEMP positively affected the classification of moderate stress. It was also observed that the partial contribution of HR (heart rate) increased.

**Figure 9:**
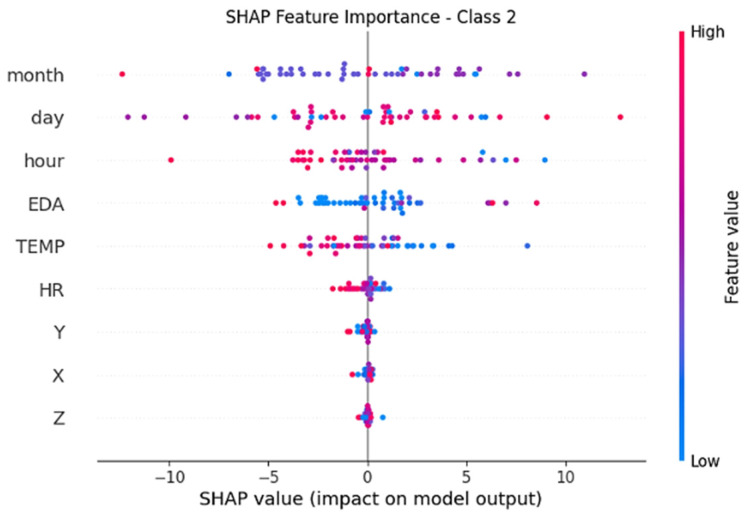
SHAP values for the TimesNet model’s Class 2 (high stress).

Figure 9 shows the attribute importance distributions of the TimesNet model over Class 2 (high stress) SHAP values. In the high stress class, the EDA and TEMP attributes are prominent. Although time factors (month, day, hour) are also effective, especially high EDA values have a strong effect on the high stress class predictions of the model. This situation supports the phenomenon that skin conductivity increases with increasing stress in accordance with physiological bases.

### 3.3. Blockchain + Chunking + Energy/Cost Analyses

In our study, a blockchain-based logging mechanism is used to ensure data security and integrity. Blockchain logs ensure that the model outputs and data chunks are recorded in an immutable manner, thereby increasing the reliability of the system and enabling retrospective verification. Table 5 shows the manifest hash, ECC signature, and timestamp information generated during the blockchain integration of the proposed system. These values confirm that each chunk is recorded reliably.

In Table 6, the effect of chunk sizes on system performance is analysed in order to protect data integrity and ensure cost-effectiveness. Since chunk size has a direct impact on verification time and retry rates, different scenarios need to be evaluated. According to Table 6, it is observed that as the chunk size decreases, the verification time increases, but the cost (calculated according to Google Cloud account costs) remains constant.

In Table 7, an ablation study was performed to evaluate the impact of blockchain-based data security and retry mechanism of the proposed system on model performance, training time, energy consumption, and cloud cost. TimesNet was taken as the base model in the study, and three separate scenarios were evaluated. In the first scenario, the model consisted solely of TimesNet. In the second scenario, TimesNet was run in conjunction with the blockchain structure. In the third scenario, a retry mechanism was used in addition to the blockchain structure to reduce losses in data transmission. In this way, the overheads and benefits of blockchain-based logging and retry approach against possible data loss are systematically analysed.

The results show that the blockchain and retry mechanisms have a negligible impact on accuracy, with the TimesNet model achieving over 99% in all scenarios. Although the training time and energy consumption increase slightly in the blockchain and retry scenarios, the difference is less than 1%. Furthermore, the cloud-based upload cost remained constant, while the retry mechanism incurred only a very low additional cost. Therefore, the blockchain and retry steps added to increase the reliability and data integrity of the system are feasible with minimal overhead in terms of performance loss or cost burden.

Table 8 extends comparison to the architectural dimension and evaluates different studies’ approaches to data integrity, explainability, and system security. According to the table, most of the studies in the literature only focus on storage efficiency or federated learning security. Chunk–RAID approaches, which provide blockchain-based integrity checking in the healthcare domain, have mostly remained at the data storage level and have not covered data transmission and model training processes. Our proposed work is the first to bridge this gap and realise chunking-blockchain integration in an edge–cloud architecture.

The proposed system provides reliable data transfer with chunk-level data integrity, ECC-based digital signature, and blockchain logging, while offering high and low energy consumption thanks to the TimesNet-based Transformer model. Furthermore, the decision process is made transparent with SHAP-based explainability analysis, and the energy and cost balance of the system is quantified with parameters such as upload time, retry cost, and GPU energy. In conclusion, this study presents a comprehensive, innovative framework that integrates partitioning, blockchain, edge–cloud collaboration, and explainable artificial intelligence into a single architecture, enabling secure data transmission and supporting transparent, reliable clinical decision-making.

## 4. Discussion and Conclusions

This paper proposes an integrated AI architecture based on cloud–edge–blockchain-chunking for IoMT-based stress detection. The results obtained show that the system differs from the existing works in the literature not only with its high accuracy rate, but also with its secure, traceable, energy-efficient and explainable structure. In particular, the integration of the chunking approach with blockchain has positioned this method, which is generally used in the literature only for data duplication or compression, as a secure data transfer and verification mechanism. In this way, the retry rate of incorrect or missing blocks during data transmission is reduced to less than 5%, and the integrity of the system is ensured by ECC signatures and manifest verifications. In terms of time series modelling, the Transformer-based TimesNet architecture outperformed other Transformer derivatives (Informer, PatchTST, NST) with its ability to capture both short-term stress fluctuations and long-term trends. SHAP-based explainability analysis made the model’s decision mechanism clinically interpretable. This is an innovative contribution of the model not only in terms of technical accuracy but also in terms of producing XAI outputs in accordance with biological foundations.

The integration of blockchain and chunking did not impose a significant performance burden on the security infrastructure of the system. The energy and cost metrics obtained show that the blockchain logging and retry mechanism increases the total energy consumption by only 0.5 per cent, while fully guaranteeing data reliability. This result shows that blockchain-chunking combination can successfully achieve a high-security/low-cost trade-off in systems with low fault tolerance, such as health data transmission. In addition, the applicability of the system to cloud–edge architecture provides a significant advantage in terms of reducing latency and maintaining energy–cost balance. When similar approaches in the literature are examined, it is seen that most of the studies focus on storage efficiency or encryption performance, but do not cover holistic factors such as data transmission, energy optimisation, explainability, and edge–cloud integration. In this respect, the proposed work is one of the first examples that combines chunking + blockchain + edge–cloud + XAI components in a single framework, enabling end-to-end secure, explainable, and cost-effective processing of IoMT data.

This study contains certain methodological limitations, and the findings should be evaluated within this framework. The Nurse Stress dataset used was obtained from a single institution, and varying sensor configurations and user behaviours across different IoMT environments may affect model performance. Energy and cost analyses were calculated via simulation rather than actual system measurements, and therefore may not fully reflect the variability seen in real-world applications. Blockchain and content-defined partitioning processes were also simulated to demonstrate the holistic functioning of the architecture. Furthermore, model validation based on fixed data separation does not fully represent situations such as sensor errors, data irregularities, and distribution changes that may occur in real IoMT flows. Future studies plan to conduct more comprehensive validation processes using real-time data obtained from multiple IoMT sources. Within this framework, the current findings support the reliability of the proposed system and the effectiveness of its integrated structure. In conclusion, this study provides a strong foundation for the development of a reliable, explainable, and energy-efficient IoMT architecture in healthcare. The proposed system is capable of pioneering the next generation of reliable AI-based healthcare systems in terms of both technical accuracy and patient safety in clinical decision support applications. With these orientations, it is aimed that the proposed system can be used not only at the academic level but also in applied health informatics systems. Especially with federated learning and smart-contract-based verification integrations, the study aims to evolve into a fully autonomous, privacy-protected and explainable digital twin infrastructure.

## Figures and Tables

**Table 1:** Overview of methods applied to the Nurse Stress dataset in the literature.

Ref.	Method	Computing Paradigm	Data Processing and Train–Test Split	Model	Performance Evaluation
[32]	TinyML-based with NearMiss-1 balancing and Min-Max normalisation	Edge (Raspberry Pi RP2040)	NearMiss undersampling, random state: 42, MinMaxScaler, train/val/test: 60/20/20	KNN	Val_Accuracy: 98% Val_Precision: 98% Val_Recall: 98% Val_F1: 98% Test_Accuracy: 98% Test_Precision: 98% Test_Recall: 98% Test_F1: 98%
[33]	Feature ranking and ROC validation	Centralised	1% subsampling for pretests, Random_state: 42, MinMaxScaler, Z-score normalisation, missing value handling, train/test: 80/20	KNN	Val_Accuracy: 90% Val_Precision: 91% Val_Recall: 90% Val_F1: 91% Test_Accuracy: 90% Test_Precision: 91% Test_Recall: 90% Test_F1: 91%
[34]	Federated learning	Cloud–edge + federated learning	Duplicate removal, Pearson correlation, timestamp, Z-score normalisation, client split: 80/20, global val/test: 50/50	Neural network	Global accuracy: 90% Precision: 85% Recall: 85% F1: 85% CL accuracy: 97% FL accuracy: 93%

**Table 2:** Model hyperparameters.

Parameter	Value
Embedding dimension	128
Layers	3
Periods	(3, 5, 7, 9, 11) (only TimesNet)
Dropout	0.1
Batch size	32
Learning rate (LR)	0.001
Optimizer	Adam
Epochs	20
Cross-validation	5 × 5 repeated stratified k-fold (25-fold)
Split	70% train, 20% validation, 10% test
Feature Selection	9

**Table 3:** Transformer-based model performance.

Model	Accuracy (%) (Mean ± STD)	Precision (%) (Mean ± STD)	Recall (%) (Mean ± STD)	F1-Score (%) (Mean ± STD)	ROC-AUC (%) (Mean ± STD)
**TimesNet**	**99.6 ± 0.08**	**99.6 ± 0.08**	**99.6 ± 0.08**	**99.6 ± 0.08**	**99.8 ± 0.13**
PatchTST	99.5 ± 0.06	99.5 ± 0.06	99.5 ± 0.06	99.5 ± 0.06	99.8 ± 0.04
TransformerEncoder	99.5 ± 0.04	99.5 ± 0.04	99.5 ± 0.04	99.5 ± 0.04	99.8 ± 0.01
Autoformer	99.3 ± 0.08	99.3 ± 0.08	99.3 ± 0.08	99.3 ± 0.08	99.7 ± 0.06
NST	99.1 ± 0.07	99.1 ± 0.07	99.1 ± 0.07	99.1 ± 0.07	99.7 ± 0.03

The model used has been highlighted in bold.

**Table 4:** Validation/test set performance of Transformer-based models.

Model	Accuracy (%) (Val/Test)	Precision (%) (Val/Test)	Recall (%) (Val/Test)	F1-Score (%) (Val/Test)	ROC-AUC (%) (Val/Test)
**TimesNet**	**99.6/99.6**	**99.6/99.6**	**99.6/99.6**	**99.6/99.6**	**99.9/99.9**
PatchTST	99.6/99.6	99.6/99.6	99.6/99.6	99.6/99.6	99.9/99.9
TransformerEncoder	99.6/99.6	99.6/99.6	99.6/99.6	99.6/99.6	99.9/99.9
Autoformer	99.4/99.4	99.4/99.4	99.4/99.4	99.4/99.4	99.8/99.8
NST	99.2/99.2	99.2/99.2	99.2/99.2	99.2/99.2	99.7/99.7

The model used has been highlighted in bold.

**Table 5:** Manifest hash, ECC signature, and timestamp generated during blockchain integration.

Parameter	Value
manifest_hash	0824bab5176126c7e3fe9be96eb20163d7018a751610c70a30d6c7b102bfeelf
ecc_signature	42248d1fd75bc733d152bb28087fbb56ea374829945ea7c05ce4e1b378cb6be
timestamp	1,759,346,521.7381518

**Table 6:** System performance of different chunk sizes.

Chunk Size	Number of Chunks	Retries	Retry Ratio	Verification Time (s)	Upload Cost (USD)
64	26,374	1318	0.049973	0.024687	0.002984
128	13,187	659	0.049973	0.013822	0.002984
256	6593	329	0.049971	0.006675	0.002984
512	3296	164	0.049757	0.003138	0.002984
1024	1648	82	0.049757	0.001657	0.002984

**Table 7:** Testing the BC + chunk architecture used in the study in different scenarios.

Scenario	Accuracy (%)	Train Time (s)	Avg. Power (W)	GPU Energy (J)	Verification Time (s)	Retry Ratio	Upload Cost (USD)
**TimesNet**	99.5	4804.817	30.178	141,831.937	0	0	0.0029
**TimesNet + BC**	99.3	4822.324	30.131	142,009.955	0.016	0	0.0029
**TimesNet + BC + Retry**	99.3	4852.261	30.062	142,642.564	0.017	0.049	0.0029

The model used has been highlighted in bold.

**Table 8:** Comparison of the proposed architecture with the studies in the literature.

Ref.	Dataset	Method	Security/Data Integrity	Model	XAI
Proposed work (Ours)	Nurse Stress (IoMT Sensor)	Chunk–SHA256–ECC, Blockchain Logging, TimesNet	Chunk-Level Integrity, ECC Signature, Blockchain Manifest	Transformer (TimesNet)	SHAP
[37]	Linux/TREC	Hybrid CDC + hash deduplication	No data integrity, compression orientated	–	–
[39]	On-chain dataset	Chunk hashing + manifest combination	SHA-256 hash + manifest validation	–	–
[40]	Health (EHR)	Chunk–RAID + AES encryption + blockchain	AES-256 + RAID integrity	Basic ML	–
[41]	Cloud storage	Adaptive compression + advanced chunking	SHA-256 + ECC validation	–	–
[60]	IoT sensor data	Blockchain + federated learning	FL parameters registered on the blockchain	Transformer-based FL	XAI

## Data Availability

The data used are publicly available and obtained from an open-access repository. Specifically, the dataset employed is the Nurse Stress Prediction from Wearable Sensors dataset, which can be accessed at the following link: P. Raval, “Nurse stress prediction using wearable sensors,” Kaggle dataset. [Online]. Available online: https://www.kaggle.com/datasets/priyankraval/nurse-stress-prediction-wearable-sensors (accessed on 15 December 2024).

## References

[1] Wang T., Lu Y., Wang J., Dai H.N., Zheng X., Jia W. (2021). EIHDP: Edge-Intelligent Hierarchical Dynamic Pricing Based on Cloud-Edge-Client Collaboration for IoT Systems. IEEE Trans. Comput..

[2] Papaioannou M., Karageorgou M., Mantas G., Sucasas V., Essop I., Rodriguez J., Lymberopoulos D. (2022). A Survey on Security Threats and Countermeasures in Internet of Medical Things (IoMT). Trans. Emerg. Telecommun. Technol..

[3] Ghubaish A., Salman T., Zolanvari M., Unal D., Al-Ali A., Jain R. (2021). Recent Advances in the Internet-of-Medical-Things (IoMT) Systems Security. IEEE Internet Things J..

[4] Manickam P., Mariappan S.A., Murugesan S.M., Hansda S., Kaushik A., Shinde R., Thipperudraswamy S.P. (2022). Artificial Intelligence (AI) and Internet of Medical Things (IoMT) Assisted Biomedical Systems for Intelligent Healthcare. Biosensors.

[5] Hederman L., Berry D., Ormazabal A. Clinician’s Perspective on Trusting Patient Generated Health Data for Use in Clinical Decision-Making: A Qualitative Interview Study. Proceedings of the 2023 IEEE Symposium on Computer-Based Medical Systems.

[6] Sun L., Sun L., Jiang X., Ren H., Guo Y. (2020). Edge-Cloud Computing and Artificial Intelligence in Internet of Medical Things: Architecture, Technology and Application. IEEE Access.

[7] Kishor A., Chakraborty C. (2022). Artificial Intelligence and Internet of Things Based Healthcare 4.0 Monitoring System. Wirel. Pers. Commun..

[8] Baygin M., Yaman O., Baygin N., Karakose M. (2022). A Blockchain-Based Approach to Smart Cargo Transportation Using UHF RFID. Expert Syst. Appl..

[9] Lakhan A., Mohammed M.A., Kozlov S., Rodrigues J.J.P.C. (2024). Mobile-Fog-Cloud Assisted Deep Reinforcement Learning and Blockchain-Enabled IoMT System for Healthcare Workflows. Trans. Emerg. Telecommun. Technol..

[10] Kai C., Zhou H., Yi Y., Huang W. (2021). Collaborative Cloud-Edge-End Task Offloading in Mobile-Edge Computing Networks with Limited Communication Capability. IEEE Trans. Cogn. Commun. Netw..

[11] Dutta J., Puthal D., Yeun C.Y. Next Generation Healthcare with Explainable AI: IoMT-Edge-Cloud Based Advanced eHealth. Proceedings of the 2023 IEEE Global Communications Conference (GLOBECOM).

[12] Dieber J., Kirrane S. (2020). Why Model Why? Assessing the Strengths and Limitations of LIME. arXiv.

[13] Marvin G., Alam M.G.R. Explainable Feature Learning for Predicting Neonatal Intensive Care Unit (NICU) Admissions. Proceedings of the 2021 IEEE International Conference on Biomedical Engineering, Computer and Information Technology for Health (BECITHCON).

[14] Arslanoğlu K., Karaköse M. Examining Patients’ Length of Stay Estimation with Explainable Artificial Intelligence Methods. Proceedings of the Fifth International Conference on Emerging Trends in Mathematical Sciences & Computing (IEMSC-24).

[15] Forti S., Ferrari G.L., Brogi A. (2020). Secure Cloud-Edge Deployments, with Trust. Future Gener. Comput. Syst..

[16] Ometov A., Molua O.L., Komarov M., Nurmi J. (2022). A Survey of Security in Cloud, Edge, and Fog Computing. Sensors.

[17] Wu Y. (2021). Cloud-Edge Orchestration for the Internet of Things: Architecture and AI-Powered Data Processing. IEEE Internet Things J..

[18] Wang X., Yang L.T., Xie X., Jin J., Jamal Deen M. (2017). A Cloud-Edge Computing Framework for Cyber-Physical-Social Services. IEEE Commun. Mag..

[19] Kar B., Yahya W., Lin Y.D., Ali A. (2023). Offloading Using Traditional Optimization and Machine Learning in Federated Cloud-Edge-Fog Systems: A Survey. IEEE Commun. Surv. Tutor..

[20] Putra K.T., Arrayyan A.Z., Hayati N., Firdaus, Damarjati C., Bakar A., Chen H.C. (2024). A Review on the Application of Internet of Medical Things in Wearable Personal Health Monitoring: A Cloud-Edge Artificial Intelligence Approach. IEEE Access.

[21] Raval  P. Nurse Stress Prediction Wearable Sensors [Data Set]. Kaggle, 2023. https://www.kaggle.com/datasets/priyankraval/nurse-stress-prediction-wearable-sensors.

[22] Dwivedi R., Mehrotra D., Chandra S. (2022). Potential of Internet of Medical Things (IoMT) Applications in Building a Smart Healthcare System: A Systematic Review. J. Oral Biol. Craniofacial Res..

[23] Kumar P., Gupta G.P., Tripathi R. (2021). An Ensemble Learning and Fog-Cloud Architecture-Driven Cyber-Attack Detection Framework for IoMT Networks. Comput. Commun..

[24] Jain S., Nehra M., Kumar R., Dilbaghi N., Hu T.Y., Kumar S., Kaushik A., Li C.Z. (2021). Internet of Medical Things (IoMT)-Integrated Biosensors for Point-of-Care Testing of Infectious Diseases. Biosens. Bioelectron..

[25] Razdan S., Sharma S. (2022). Internet of Medical Things (IoMT): Overview, Emerging Technologies, and Case Studies. IETE Tech. Rev..

[26] Liu W., Zhao F., Shankar A., Maple C., Peter J.D., Kim B.G., Slowik A., Parameshachari B.D., Lv J. (2025). Explainable AI for Medical Image Analysis in Medical Cyber-Physical Systems: Enhancing Transparency and Trustworthiness of IoMT. IEEE J. Biomed. Health Inform..

[27] Rani S., Kataria A., Kumar S., Tiwari P. (2023). Federated Learning for Secure IoMT-Applications in Smart Healthcare Systems: A Comprehensive Review. Knowl-Based Syst..

[28] Ausín J.L., Ramos J., Lorido A., Molina P., Duque-Carrillo J.F. (2023). Wearable and Noninvasive Device for Integral Congestive Heart Failure Management in the IoMT Paradigm. Sensors.

[29] Ding C., Zhou A., Liu Y., Chang R.N., Hsu C.H., Wang S. (2022). A Cloud-Edge Collaboration Framework for Cognitive Service. IEEE Trans. Cloud Comput..

[30] Wu Q., Chen X., Zhou Z., Zhang J. (2020). FedHome: Cloud-Edge Based Personalized Federated Learning for In-Home Health Monitoring. IEEE Trans. Mob. Comput..

[31] Gao Z., Zhang H., Dong S., Sun S., Wang X., Yang G., Wu W., Li S., De Albuquerque V.H.C. (2020). Salient Object Detection in the Distributed Cloud-Edge Intelligent Network. IEEE Netw..

[32] Abu-Samah A., Ghaffa D., Abdullah N.F., Kamal N., Nordin R., Dela Cruz J.C., Magwili G.V., Mercado R.J. (2025). Deployment of TinyML-Based Stress Classification Using Computational Constrained Health Wearable. Electronics.

[33] Chauhan R., Singh D. (2025). Predictive Analytics for Stress Management in Nursing: A Machine Learning Approach Using Wearable IoT Devices. Lect. Notes Comput. Sci..

[34] Liu K., Xue W., Hou D. (2025). Federated Learning for Nurse Stress Prediction Using Wearable Sensors: Integrating Biomechanical Data. Mol. Cell. Biomech..

[35] Feng D. (2022). Chunking Algorithms. Data Deduplication in High Performance Storage Systems.

[36] Ruba S., Kalpana A.M. (2025). Advanced Chunk-Based Data Deduplication Framework for Secure Data Storage in Cloud Using Hybrid Heuristic Assisted Optimal Key-Based Encryption. Wirel. Netw..

[37] Arora R., Vetrithangam D. SmartChunk: A Hybrid Content-Based Chunking Algorithm with Hash De-Duplication for Effective Data Deduplication in Cloud Storage System. Proceedings of the 1st International Conference on Innovative Communication and Electrical and Computer Engineering (ICICEC).

[38] Onmalwar V.M., Vinoth Kumar C.N.S. (2025). Cloud-Based Encryption and Chunking for Data Management. Lect. Notes Netw. Syst..

[39] Tmeizeh M., Rodríguez-Domínguez C., Hurtado-Torres M.V. (2024). File Chunking Towards On-Chain Storage: A Blockchain-Based Data Preservation Framework. Clust. Comput..

[40] Babu S.B., Jothi K. (2024). A Robust Model for a Healthcare System with Chunk-Based RAID Encryption in a Multitenant Blockchain Network. Int. J. Adv. Comput. Sci. Appl..

[41] Tmeizeh M., Rodríguez-Domínguez C., Hurtado-Torres M.V. (2025). Optimizing Blockchain File Storage: Enhancing Performance and Reducing Ledger Size with Adaptive Compression and Advanced Data Structures. Clust. Comput..

[42] Pusztai T., Nastic S. (2025). ChunkFunc: Dynamic SLO-Aware Configuration of Serverless Functions. IEEE Trans. Parallel Distrib. Syst..

[43] Hua H., Li Y., Wang T., Dong N., Li W., Cao J. (2023). Edge Computing with Artificial Intelligence: A Machine Learning Perspective. ACM Comput. Surv..

[44] Bao G., Guo P. (2022). Federated Learning in Cloud-Edge Collaborative Architecture: Key Technologies, Applications and Challenges. J. Cloud Comput..

[45] Pham C., Nguyen D.T., Njah Y., Tran N.H., Nguyen K.K., Cheriet M. (2022). Share-to-Run IoT Services in Edge Cloud Computing. IEEE Internet Things J..

[46] Fang J., Ma A. (2021). IoT Application Modules Placement and Dynamic Task Processing in Edge-Cloud Computing. IEEE Internet Things J..

[47] Guo M., Li L., Guan Q. (2019). Energy-Efficient and Delay-Guaranteed Workload Allocation in IoT-Edge-Cloud Computing Systems. IEEE Access.

[48] Haji L., Ahmed O., Dino H., Haji L.M., Ahmad O.M., Zeebaree S.R.M., Dino H.I., Zebari R.R., Shukur H.M. (2020). Impact of Cloud Computing and Internet of Things on the Future Internet. Technol. Rep. Kansai Univ..

[49] Dang L.M., Piran M.J., Han D., Min K., Moon H. (2019). A Survey on Internet of Things and Cloud Computing for Healthcare. Electronics.

[50] Anikwe C.V., Nweke H.F., Ikegwu A.C., Egwuonwu C.A., Onu F.U., Alo U.R., Teh Y.W. (2022). Mobile and Wearable Sensors for Data-Driven Health Monitoring System: State-of-the-Art and Future Prospect. Expert Syst. Appl..

[51] Angel N.A., Ravindran D., Vincent P.M.D.R., Srinivasan K., Hu Y.C. (2022). Recent Advances in Evolving Computing Paradigms: Cloud, Edge, and Fog Technologies. Sensors.

[52] Bacanin N., Zivkovic M., Bezdan T., Venkatachalam K., Abouhawwash M. (2022). Modified Firefly Algorithm for Workflow Scheduling in Cloud-Edge Environment. Neural Comput. Appl..

[53] Chen X., Zhang J., Lin B., Chen Z., Wolter K., Min G. (2022). Energy-Efficient Offloading for DNN-Based Smart IoT Systems in Cloud-Edge Environments. IEEE Trans. Parallel Distrib. Syst..

[54] Zhao X., Cheng S., Lu G., Zhou H., Jia B., You Y. AutoChunk: Automated Activation Chunk for Memory-Efficient Long Sequence Inference. Proceedings of the 12th International Conference on Learning Representations (ICLR).

[55] Le K., Ho T.V., Tran D., Chau D.T. ChunkFormer: Masked Chunking Conformer for Long-Form Speech Transcription. Proceedings of the 2025 IEEE International Conference on Acoustics, Speech and Signal Processing (ICASSP).

[56] Mourtzis D., Angelopoulos J., Panopoulos N. (2023). Blockchain Integration in the Era of Industrial Metaverse. Appl. Sci..

[57] Tan T.M., Saraniemi S. (2023). Trust in Blockchain-Enabled Exchanges: Future Directions in Blockchain Marketing. J. Acad. Mark. Sci..

[58] Han H., Shiwakoti R.K., Jarvis R., Mordi C., Botchie D. (2023). Accounting and Auditing with Blockchain Technology and Artificial Intelligence: A Literature Review. Int. J. Account. Inf. Syst..

[59] Gousia H., Sparsh S., Sara I., Imtiaz A., Shaima Q., Malik I. (2022). Blockchain Technology: Benefits, Challenges, Applications, and Integration of Blockchain Technology with Cloud Computing. Future Internet.

[60] Huynh-The T., Gadekallu T.R., Wang W., Yenduri G., Ranaweera P., Pham Q.-V., da Costa D.B., Liyanage M. (2023). Blockchain for the Metaverse: A Review. Future Gener. Comput. Syst..

[61] Guo H., Yu X. (2022). A Survey on Blockchain Technology and Its Security. Blockchain Res. Appl..

[62] Seok B., Park J., Park J.H. (2019). A Lightweight Hash-Based Blockchain Architecture for Industrial IoT. Appl. Sci..

[63] Devika K.N., Bhakthavatchalu R. Parameterizable FPGA Implementation of SHA-256 Using Blockchain Concept. Proceedings of the 2019 IEEE International Conference on Communication and Signal Processing (ICCSP).

[64] Wang Q., Yang Y., Zhao M., Wan H., Li B., Yan X. (2025). Chaotic Parallel Hash Engine with Dynamic Stochastic Diffusion for Blockchain and Cloud Security. Sci. Rep..

[65] Du Z., Pang X., Qian H. (2021). PartitionChain: A Scalable and Reliable Data Storage Strategy for Permissioned Blockchain. IEEE Trans. Knowl. Data Eng..

[66] Bera S., Dey T., Mukherjee A., Bhattacharya P., De D. (2025). FedChain: Decentralized Federated Learning and Blockchain-Assisted System for Sustainable Irrigation. IEEE Trans. Consum. Electron..

[67] Vaswani A., Brain G., Shazeer N., Parmar N., Uszkoreit J., Jones L., Gomez A.N., Kaiser Ł., Polosukhin I. Attention Is All You Need. Proceedings of the Advances in Neural Information Processing Systems 30 (NIPS 2017).

[68] Zhou H., Zhang S., Peng J., Zhang S., Li J., Xiong H., Zhang W. Informer: Beyond Efficient Transformer for Long Sequence Time-Series Forecasting. Proceedings of the AAAI Conference on Artificial Intelligence 2021.

[69] Akuthota U.C., Bhargava L. (2025). Transformer-Based Intrusion Detection for IoT Networks. IEEE Internet Things J..

[70] Wu H., Hu T., Liu Y., Zhou H., Wang J. (2022). TimesNet: Temporal 2D-Variation Modeling for General Time Series Analysis. arXiv.

[71] ElkanaEbinazer S. (2025). Hybrid Encryption with Greylag Goose Optimizer-Based Swin Transformer for Secured Data Deduplication and Anomaly Detection for Cloud-Based IoMT Applications. SSRN.

[72] Jayakarthik R., Suneel S., Thatipudi J.G., Jaganraja V., Vohra M., Gopinath D. FogMedX-Transform: A Transformer-Based Task Interoperability Framework for Energy-Efficient Fog-Enabled IoMT. Proceedings of the 8th Innovative Computing Technologies (ICICT).

[73] Kalakoti R., Nomm S., Bahsi H. Explainable Transformer-Based Intrusion Detection in Internet of Medical Things (IoMT) Networks. Proceedings of the International Conference on Machine Learning and Applications (ICMLA).

